# Distribution and Evolutionary History of the Mobile Genetic Element s2m in Coronaviruses

**DOI:** 10.3390/diseases4030027

**Published:** 2016-07-28

**Authors:** Torstein Tengs, Christine M. Jonassen

**Affiliations:** 1 Norwegian Veterinary Institute, Ullevaalsveien 68, 0454 Oslo, Norway; 2 Centre for Laboratory Medicine, Østfold Hospital Trust, Kalnesveien 300, 1714 Grålum, Norway; chrjon@so-hf.no

**Keywords:** s2m, coronavirus, secondary structure, mobile genetic element

## Abstract

The mobile genetic element s2m has been described in several families of single-stranded RNA viruses. The function remains elusive, but an increasing number of s2m-containing sequences are being deposited in publicly available databases. Currently, more than 700 coronavirus sequences containing s2m can be found in GenBank, including the severe acute respiratory syndrome (SARS) coronavirus genome. This is an updated review of the pattern of s2m in coronaviruses, the possible functional implications and the evolutionary history.

## 1. Introduction

The genomes of single-stranded RNA (ssRNA) viruses have complex secondary structures, maintained both through conventional and non-Watson-Crick base-pairing [1,2]. Coronaviruses have the largest genomes of RNA viruses and have been shown to have several highly conserved secondary structures both in the 5′ and 3′ end of their genomes [1]. These structural motifs mainly function through protein binding or through direct RNA-RNA interactions. Some of these elements function merely as ‘spacer elements’, where neither the primary nor the secondary structure are critical [3]. In other cases, the stem-loop structure itself seems essential and base-pairing nucleotides may be substituted [4]. More complex structures, such as the 54-nucleotide hairpin-type pseudoknot, appear to be conserved both in structure and location, but not in sequence [5]. For most of these elements, the exact mechanism of function is unknown, but virus replication, viability and transcription are the main functional categories.

A 43-nucleotide genetic element with a highly conserved secondary (as well as primary and tertiary) structure has been described in four different families of positive-sense ssRNA viruses, *Astroviridae*, *Caliciviridae*, *Picornaviridae* and *Coronaviridae* [6]. The presence of this element in four distantly related groups of viruses and the scattered distribution within these groups has led to the conclusion that viruses have the ability to acquire it horizontally [6]. The 3′ stem-loop structure, referred to as s2m, was originally described in astroviruses where it appears to have been present in the last common ancestor [6,7,8], but the element has been most thoroughly studied in the SARS coronavirus (SARS-CoV) where the three-dimensional crystal structure has been resolved to 2.7 A resolution [9]. The function remains obscure, but current hypotheses include hijacking of host protein synthesis through interactions with ribosomal proteins [9] and RNA interference (RNAi) via processing of the s2m elements into a mature microRNA [6]. In coronaviruses, the highly conserved nature of the element has also allowed the development of a PCR-based virus discovery strategy [10]. 

The presence of s2m near the 3′ end of some coronavirus genomes has been documented previously [6,11], but due to the exponential growth of gene sequence data available through public databases, this review is an update on the current status of s2m in this virus family. The evolutionary history is discussed and different hypotheses for the possible function of s2m are presented in light of recent progress in virus genomics.

## 2. Coronavirus Phylogeny and Distribution of s2m

**Figure 1:**
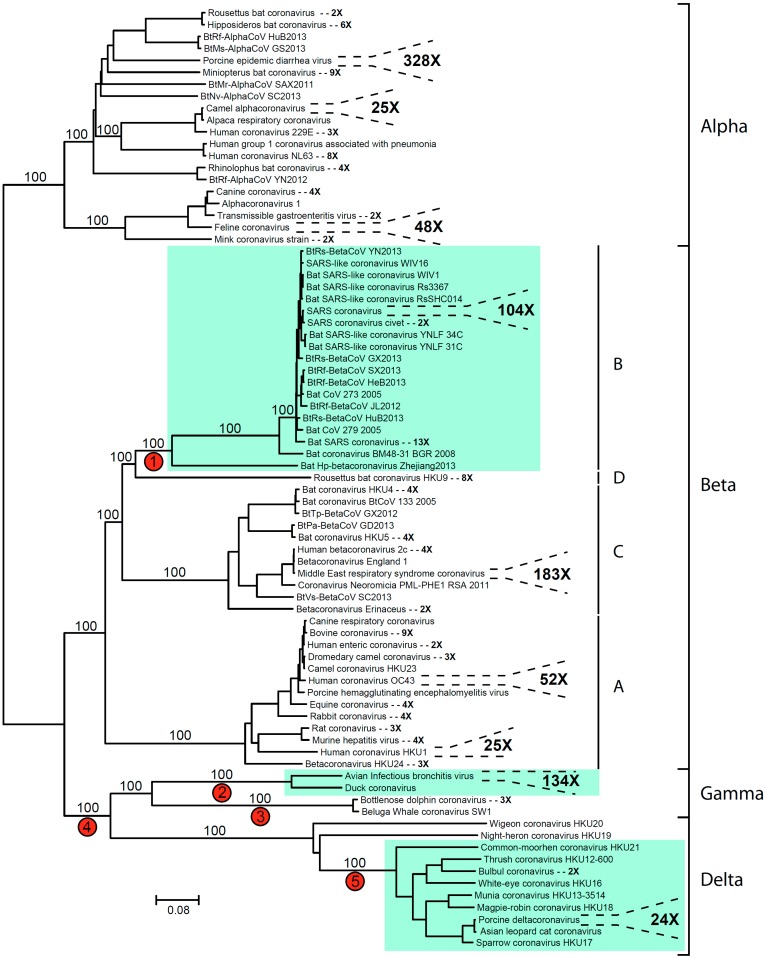
Coronavirus phylogeny with s2m-containing sequences highlighted. ORF1ab polyprotein amino acid sequences were aligned using the program MUSCLE [8] and default parameters. The phylogenetic analysis was performed using the program SeaView [9] and the neighbor joining clustering method with Kimura two-parameter distances. In order to avoid large clades of closely related sequences, operational taxonomic units (OTUs) with similar GenBank taxonomical annotation and almost identical sequences were identified and basal members of these monophyletic groups chosen to represent such sequence clusters. For instance, there are 183 complete ORF1ab polyprotein sequences available from different strains of the Middle East respiratory syndrome (MERS) coronavirus. These sequences are represented by a single accession (in this case, GenBank accession number ALB08298; isolate KOREA/Seoul/035-1-2015). Based on visual inspection of the alignment it was determined that the sequences belonging to the Torovirinae subfamily could not be reliably aligned and were excluded from the analysis. Brackets show serogroups as well as betacoronavirus lineages and key branches with 100% bootstrap support (100 pseudoreplicates) have been indicated. Red circles indicate possible losses/gains of s2m (see discussion in text).

A total of 20,068 coronavirus nucleotide sequences have been deposited in the current version of Genbank, and when translated into amino acid sequences, the complete ORF1ab polyprotein sequence could be found for 1113 of these entries. A phylogenetic analysis was performed using aligned ORF1ab sequences (Figure 1), and numerous clusters of nearly identical accessions could be identified. The largest group comprised 328 sequences from the porcine epidemic diarrhea virus (328 OTUs). Many smaller clusters were also found, encompassing two to 183 sequences, and all serogroups were monophyletic with 100% bootstrap support (Figure 1).

To get an overview of the current status for s2m in coronaviruses, all coronavirus nucleotide sequences were screened for the presence of the s2m motif using the strategy described by Tengs et al. [6]. The consensus sequence CGNGG(N)CCACGNNGNGT(N)ANNANCGAGGGT(N)ACAG was used as bait, allowing for possible insertions (N) and/or a single mismatch. Further, 708 of 20,068 (3.5%) of the sequences were found to contain the s2m motif (Table 1). However, s2m was not found in alpha coronaviruses and also appears to be absent in the *Torovirus* genus as well as the recently described *Bafinivirus* genus [12], albeit only a small number of sequences were available from the two latter groups. In all instances, the s2m sequence was found in the non-coding, 3′ end of the genome. Looking at coronavirus accessions annotated as ‘complete genome’, more than a third of the genomes appeared to be s2m-containing (Table 1). Many sequences have been generated using targeted (PCR-based) approaches that might not include the non-coding parts of the genome. This could explain why s2m appears to be approximately 10 times more common in complete genome sequences than in shorter GenBank submissions (Table 1).

Three phylogenetic clusters were found to contain s2m (Figure 1). All of these groups were supported by 100% bootstrap values and thus are genetically distinct from related viruses not containing the element. Multiple hypothesis can be proposed in order to explain the distribution of s2m within the coronaviruses, including a model where s2m was present in the last common ancestor of this virus group and subsequently lost multiple times. However, given the mobile nature of the motif, a more parsimonious explanation includes two (or more) independent gains and a small number of losses. The gain of s2m at the base of the SARS-cluster (edge 1 in Figure 1) would not assume any losses in the beta coronaviruses, but for the gamma/delta coronavirus cluster, the evolutionary history seems to be more complicated. A gain at edge 4 would require multiple independent losses. There was no significant bootstrap support separating the Wigeon coronavirus and the Night-heron coronavirus sequences, so a monophyletic origin for these two strains could not be excluded. Loss in the common ancestor of this group combined with loss prior to the divergence of the Bottlenose dolphin coronavirus and the Beluga Whale coronavirus (edge 3) would thus indicate one gain and two independent losses. One could also hypothesize two independent gains (edge 2 and 5) in order to explain the s2m distribution in the gamma/delta cluster (Figure 1).

Looking at topology and branch lengths, branch 1 (Figure 1) represents the shortest edge connecting an s2m-containing cluster with a virus strain not containing s2m. s2m is absent in Rousettus bat coronavirus HKU9, which is a cluster of eight nearly identical sequences basal to the SARS(-like) group. Rousettus bat coronavirus is thus far the only member of lineage D within the betacoronaviruses [13]. The most likely mechanism for transfer of the s2m element from one viral genome to another is through genetic recombination [6]. Recombination events are very common in single-stranded RNA viruses and have played an important role in the evolution of coronaviruses [14]. Nucleotide dot plot analyses of the Rousettus bat coronavirus versus the closest relatives in the SARS(-like) group revealed a high degree of sequence similarity in central parts of the genome, but did not identify any specific locus for recombination.

## 3. Sequence and Secondary Structure of s2m

**Figure 2:**
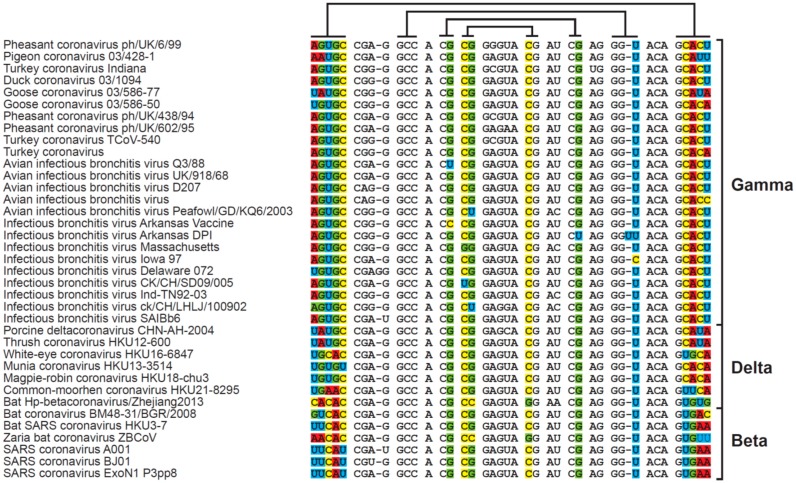
s2m coronavirus sequence motifs. For each genotype, one representative accession was chosen to illustrate the conserved nature of s2m both on a primary (‘sequence’) level and on a secondary (stem-loop structure) level. Lines above alignment indicate co-varying/stem-forming elements and columns with non-conserved bases for these nucleotide positions have been color coded.

**Figure 3:**
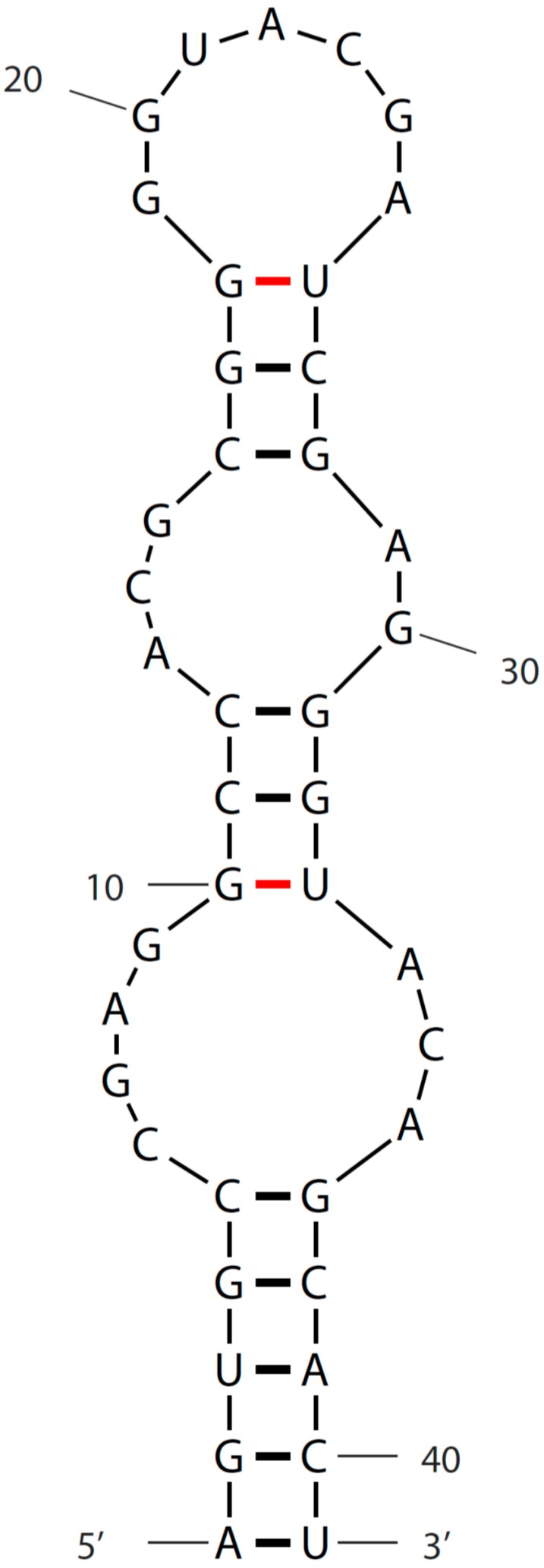
s2m secondary structure. The s2m element from Pheasant coronavirus strain ph/UK/6/99 was folded using mfold [16]. Non-Watson-Crick base-pairings are shown in red. For a more detailed folding with tertiary interactions and long-range contacts indicated, see [9].

Excluding sequences with ambiguous bases, a total of 37 different s2m genotypes were found within the coronavirus sequences (Figure 2). The great majority of the sequences could be folded into the canonical s2m stem-loop structure (Figure 3), albeit a single sequence had an insertion in the second stem-forming motif (Figure 1; Infectious bronchitis virus DPI) and some of the sequences require non-Watson-Crick base-pairing, such as the wobble base-pair G-U [15], in order to obtain the correct secondary structure. All clusters of identical s2m sequence groups were derived from viruses representing the same serogroups (beta, gamma or delta), indicating no recent transfers of the s2m element between these coronavirus clusters.

## 4. Function of s2m

For a mobile genetic element to be successful, especially in the context of rapidly evolving genomes such as those of RNA viruses, it must offer an immediate selective advantage. s2m viruses infect higher vertebrates, making the permissive host species a lot more closely related than the viruses. The observation that s2m can apparently be transferred between unrelated viruses and remain functional (under selection to maintain sequence and structure) suggests strongly that the target for s2m is host-specific and not viral. As there are no conserved elements flanking the s2m motif in viral genomes, it is also plausible that the element has an ‘autonomous’ function, independent of other viral genes, transcripts or genome secondary/tertiary structures. Somewhat surprisingly, the acquisition of s2m only has a subtle effect; exchanging the (non-s2m-containing) 3′-end of a murine coronavirus (MCV) with the 3′-end of a SARS virus did not appear to have any dramatic consequences [17], but 3′-ends from other coronaviruses were not able to replace the 3′-end of MCV and render it viable. There appears to be only a single sequence in GenBank with a deleted version of s2m (Infectious bronchitis virus strain ck/CH/LHLJ/07VII; accession number JF274479). This strain was discovered as an escape mutant in a vaccine development project, but did not appear phenotypically different from closely related viruses in culturing experiments [18].

The folding of s2m is quite similar to the hairpin structures formed by microRNAs that are involved in RNAi-associated gene regulation, and s2m could potentially be involved in gene silencing. In vertebrates, pre-microRNA transcripts are generated in the nucleus and processed by the nuclear protein DGCR8 and the enzyme Drosha [19]. Processed pre-microRNAs are then exported from the nucleus in a process involving Exportin-5. The RNA is subsequently incorporated into the RNA-induced silencing complex (RISC) and eventually a functional RISC-bound small interfering RNA (siRNA) can serve as template for base-pairing recognition of messenger RNA (mRNA). The target mRNA is degraded, leading to a reduction in gene activity. ssRNA viruses generally replicate in the cytoplasm and earlier hypotheses on s2m function have thus been based on the assumption that the cellular components involved must be available in the cytosol. Recently, a paper was published documenting the presence of a non-canonical machinery for microRNA processing in the cytoplasm of human cells [20]. It has been well described that DNA viruses replicating in the nucleus can hijack the cellular machinery for RNAi activity for the regulation of both viral and host genes [21], but these new results using a recombinant Sindbis virus show that this is also possible for RNA viruses. The presence of multiple copies of s2m in some virus genomes [6] can be explained by there being an additive effect: more copies of the RNA will enable the formation of more siRNA/RISC complexes and give a more profound effect on target genes. A hypothesis where s2m is involved in RNAi-based gene regulation is also compatible with the target being host-specific (homologous genes in the infected species) and s2m functionality being independent of the rest of the virus genome.

Observations arguing against an RNAi-like function would be that the stem region of s2m is a bit short compared to standard processed RNAi molecules (albeit mRNA binding regions as short as 17 base pairs have been reported [22]). We were also unable to find any good candidate target genes using sequence similarity searches to look for potential microRNA binding sites, and even when looking at viruses infecting relatively closely related species (such as bats, see Figure 2), there was significant variability in the s2m stem regions that could be predicted to be involved in mRNA binding. These observations make it less likely that s2m is involved in conventional RNAi-based gene silencing.

Another hypothesis that would be compatible with the general assumptions regarding s2m functionality is that s2m is involved in the protection of the virus genome from being degraded by host ribonucleases. Coronaviruses are positive-sense ssRNA viruses with non-segmented genomes that are polyadenylated and capped. The genomes may serve as mRNA for the translation of viral polyproteins and contain two overlapping reading frames that encode precursor polyproteins pp1a and pp1ab through a frameshifting mechanism [23]. After infection, the viral genome is replicated and subgenomic RNAs are generated via negative-strand RNA intermediates [1,24]. In flaviviruses, another group of positive-sense ssRNA viruses, the presence of secondary structure elements has been shown to inhibit RNA decay through the formation of nuclease-resistant noncoding RNAs [25,26]. The small stem-loop structures described confer resistance to enzymatic degradation by the XRN1 5′-3′ exoribonuclease. As the s2m element is near the 3′ end of the coronavirus genome, inhibition of, for instance, XRN1 would only render the negative-strand versions of the genome (or subgenomic elements originating from the 3′ flank) protected from decay, but resistance could also be gained through other mechanisms. Full-length coronavirus messenger RNAs share many features with endogenous transcripts and any interference with mechanisms involved in cellular mRNA turnover could also affect virus RNA stability. The exosome contains multiple proteins involved in 3′-5′ degradation of RNA [27], and inhibition or stalling of, for instance, the Dis3 or Rrp6 polypeptides by s2m could also protect full-length sense copies of the genome.

For some positive-strand ssRNA viruses, genome circularization has been shown to be essential for virus replication [28]. RNA circularization would require s2m to bind either directly or indirectly (mediated by RNA binding proteins) to the 5′ end of the genome. The primary binding 5′ site could either be a five-prime cap (5′ cap) or an RNA structure (see discussion in [29]). It is possible that there are conserved structural RNA elements with such a binding affinity near the 5′ end in all s2m viruses, but it seems unlikely given the complete lack of sequence similarity when comparing data from the 5′ flank of these genomes. The direct involvement of a 5′ cap also seems implausible, as, for instance, s2m-containing picornaviruses lack 5′ capping.

In order to elucidate the function of s2m, it seems imperative to establish a reverse genetics system for an s2m-containing viral strain. A good system could be an astrovirus-derived infectious construct, as there already are protocols described for the design of astrovirus cDNA clones that lead to the production of infectious viral particles [30]. Targeted mutations may be introduced and comparative studies performed using different permissive cell lines. Any changes in transcriptional activity, virus replication rates and/or viral RNA stability can be correlated with mutations interfering with the structure of s2m. Though s2m is present in some coronaviruses associated with severe infections in humans, such as SARS-CoV, there are other highly pathogenic viruses where s2m is absent (for instance the Middle East respiratory syndrome coronavirus; MERS-CoV). The significance of having s2m for a viral strain remains to be resolved, but this mobile element still seems to be in play and it is likely to be found in newly emerging coronaviruses in the future.

## Figures and Tables

**Table 1:** Coronavirus sequences in GenBank.

	Total	Containing s2m
Coronavirus sequences	20,068	706 (3.5%)
Alpha coronavirus *	7190	0
Beta coronavirus *	4947	342 (6.9%)
Delta coronavirus *	141	60 (42.6%)
Gamma coronavirus *	6360	281 (4.4%)
Bafinivirus *	12	0
Torovirus *	307	0
Complete genomes	1507	523 (34.7%)

* GenBank taxonomy database annotation.
